# Childhood trauma and other formative life experiences predict environmental engagement

**DOI:** 10.1038/s41598-022-24517-7

**Published:** 2022-12-01

**Authors:** Urooj S. Raja, Amanda R. Carrico

**Affiliations:** 1 School of Communication, Loyola University Chicago, Chicago, IL USA; 2 Department of Environmental Studies, The University of Colorado Boulder, Boulder, CO, USA

**Keywords:** Psychology, Environmental social sciences

## Abstract

Environmental problems continue to intensify. Yet, despite scientific consensus on threats such as climate change, broadscale public engagement with the issue is elusive. In this paper, we focus on childhood formative experiences and the extent to which they are correlated with environmental engagement. We consider two forms of environmental engagement: civic engagement, measured in hours per month devoted to an environmental protection cause, and private-sphere green behavior. Past studies about significant life experiences have shown that formative experiences, especially in childhood, correlate with environmentally sensitive attitudes and vocations in later life. However, we know less about the formative life events experienced by contemporary environmentally engaged persons. Looking at a nationally representative sample of American adults (n = 449), we find that childhood trauma predicts both civic engagement and green behavior. We also find that childhood experiences in nature and childhood travel experiences predict green behavior but not civic engagement.

## Introduction

Despite the urgency of contemporary environmental problems, the number of people currently engaged in environmental protection is inadequate. In 2021, Pew researchers conducted a national survey in which they found that only 24% of U.S. adults had engaged in political action to address climate change (e.g., monetary donation, contacting an elected official) in the past year^1^. Though we know much about the barriers to environmental engagement, we know less about the drivers of engagement. What drives the minority of people who are engaged? In this analysis, we examine how formative or early life experiences are associated with environmental engagement in adulthood^2–4^. In doing so, we attempt to draw connections between existing research demonstrating the significance of early life experiences in the formation of environmental values^2,3,5,6^ and the larger literature examining factors associated with pro-environmental behavior in adulthood^7–11^. Environmental engagement is a broad term. Several scholars define the term as involving the adoption of pro-environmental actions^5,12,13^. Others employ a broader definition that includes pro-environmental behavioral intent^14^, climate policy support^15^, and environmental concern^16^. Paul Stern and group identify two spheres of action: public and private. These include public sphere behaviors like writing letters to elected officials, donating time and resources to movement organizations, or learning about movement organizations^17^. Private sphere behaviors describe those adopted by individuals or households to reduce environmental impact (e.g., recycling, reducing airline travel). In the analysis described below, we examine the relationship between early life experiences and both forms of environmental engagement.

### Formative experiences and environmental engagement

The Significant Life Experiences (SLE) Framework aims to understand how formative experiences affect attitudes, values, and behavior in later life^2,3,6,18,19^. SLE researchers chiefly study environmental protection workers, educators, and volunteers^2,6,18^. Much of the seminal SLE work has been qualitative and draws on extensive life course interviews^2,3,5^. Significant life experiences may include childhood experiences in nature (e.g., interacting with wildlife, hiking), travel experiences, interacting with role models, participating in nature-based educational activities, or reading formative books^2,18,20^. Formative experiences are often emotionally powerful, which may make it easier for people to recall these experiences in later life^21^. Formative experiences are often novel, or what some researchers^2^ describe as those that create a “first bond” with nature. For example, a person swimming in a lake for the first time. Novel experiences are likely to occur in childhood^2^. Evidence also suggests that formative experiences may allow for play^2,22^ and may involve social interaction, especially with family and friends ^2,23^.

### Experiences in nature

Researchers have found a particularly strong link between childhood experiences in nature and pro-environmental attitudes and behaviors in later life^2,19,24–26^. In a seminal SLE study, Louise Chawla interviewed 30 self-identified environmentalists from Kentucky and Norway and asked these respondents to relay the story of why they became involved with environmental work^2^. Environmentalists spoke about their childhood experiences in nature as instrumental to their decision to become environmentalists. For example, participants talked about long periods spent outdoors as a child, fond moments in childhood gardens, or traversing hiking trails as children. Evans and colleagues (2018) also conducted a longitudinal study^11^. They measured how much time 99 children spent outdoors. They followed up with 74 of these children 12 years later when they were 18 years of age and found that the children who had spent more time outdoors were more likely to exhibit pro-environmental behaviors than counterparts who had not spent time outdoors.

### Travel experience

Travel experience is also an important precursor to environmental engagement within the SLE literature. In one study, a researcher interviewed 16 people committed to environmental change at a public university in California and found that travel, especially in the form of study abroad, influenced environmental action^27^. The researchers found that travel is especially transformative because people often go to places outside their comfort zone, where they must adapt and learn about new things. For example, one respondent said while traveling, she learned about a farm supplying fresh food to a community. This motivated her to think about how such services could run in her local area. Travel also compels people to develop an awareness of global issues and motivates them to transcend cultural boundaries and borders^28,29^. As a result, it may be easier for such persons to grasp social and environmental problems, such as climate change, which transcend local boundaries.

### Trauma as a child

Even though SLE researchers have identified childhood experiences as central, they generally have not examined the effect of adverse childhood experiences on later environmental engagement. A few scholars in the qualitative tradition have remarked that negative experiences about the environment do show up in life-case interviews with environmentalists. For example, in one study, researchers interviewed youth activists. They found that some had experienced climate devastation from hurricanes and floods, which they later recounted as contributing to their engagement trajectory^30^. Other researchers have also interviewed environmentalists and found that their experiences observing habitat destruction earlier in their life affected their environmental commitments in later life^2^. Prior exploratory work by these authors also revealed a possible connection between trauma as a child and environmental engagement (BLIND, in prep). In a series of qualitative interviews, participants spoke about childhood trauma as being instrumental in their dedication to environmental protection work. Specifically, participants said they felt empathy toward the environment, which was also suffering.

Quantitative work focusing specifically on the relationship between trauma and pro-social behavior is relatively rare; however, some interesting findings have emerged. For example, Kaniasty and Norris (1995) found that people who experienced a collective trauma were more likely to engage in pro-social behavior than nonvictims^31^. Studies also show that victims help similar victims but not nonvictims, in part because they may feel responsible for preventing suffering in similar others^32–34^. Other studies reveal positive associations between trauma and pro-social behaviors, for example, volunteering and helping others^31–33,35^. This latter work has found that the propensity to engage in pro-social outcomes could be explained by the fact that people experience empathy and compassion for the aggrieved other^35^, are resilient^36^, are attempting to find meaning in something larger, or are attempting to help themselves by helping another^32,33^.

With regards to the positive association between personal trauma and empathy^34,37–39^, Greenberg and colleagues found that adults who had experienced a childhood trauma scored higher on empathy than adults who did not^34^. Here childhood trauma was measured using the childhood traumatic events scale, which asks questions about a range of adverse effects such as the death of a family member and sexual violence. The authors postulate that traumatic events cause people to become more emotionally aware and attuned to cues that signal distress. This is the same logic behind support groups, in which the assumption is that participants are more likely to listen, understand, and react in ways that reflect their shared experiences. Evidence also suggests a positive link between empathy and pro-environmental behavior. For example, scholars have looked at the helping behaviors of 60 students from Madrid^40^. In this study, researchers assigned participants to high and low empathy conditions. In the high empathy condition, a prompt encouraged participants to take the perspective of a bird in an oil spill or cut trees on the ground. In the low empathy condition, the prompt encouraged participants to adopt an objective stance when viewing either the bird or tree image. Participants in the high empathy condition manifested stronger environmental behavior. Scholars have found a similar pattern among Chinese undergraduates in that empathy moderately but positively correlated with green behavior^41^. Here, participants were asked questions about protective feelings towards non-human nature, for example, plants and animals.

Importantly, evidence also suggests that experiencing childhood trauma is associated with aggression, drug use, criminal pathology, and suicide ideation^42,43^. For example, one cluster of studies found negative associations between experiencing trauma and pro-social behavior measured in the form of cognitive, affective, and behavioral attitudes towards altruism^43,44^, though in non-representative samples^44^. As such, we might also expect childhood trauma to be associated with less environmental concern or pro-environmental behavior. Yet, research on childhood trauma focuses disproportionately on negative outcomes, and few studies have examined relationships between past traumatic experiences and care for the environment^32,33,36,43,44^. Trauma is also highly variable and contextual, and^32,36^; as such, we need more information on how it operates in relation to environmental engagement.

### Research gaps and study objectives

Although the SLE literature suggests a strong connection between childhood experiences and environmental behavior in adulthood, the bulk of this work is qualitative and based on small non-representative samples limiting its generalizability^45^. Among the existing quantitative studies, most have been done outside the United States (see, for example^20,24^). Likewise, although some SLE work also considers attitudes and private-sphere behavior in adulthood, we know significantly less about public-sphere behavior^22,24^.

This study addresses these gaps by analyzing quantitative survey data from a national sample of American adults. Specifically, we examine the relationship between childhood SLEs and environmental engagement in the form of public sphere engagement (termed here *civic engagement*) and private sphere behavior (*green behavior*) during adulthood. Building on existing literature, we consider SLE’s in the form of experiences in nature and travel experiences. We further advance existing work by also considering childhood trauma, which has received substantially less attention within the existing literature. We also note that our selection of variables was, in part, inspired by another qualitative study in which participants spoke about the significance of the three formative experiences above as being instrumental in their dedication to environmental protection work (BLIND, in prep).

In summary, this study examines two questions: Are SLE’s (e.g., experiences in nature, travel experiences, and childhood trauma) correlates of (1) civic engagement and (2) green behavior in a sample of American adults? We predict that exposure to these SLEs will be associated with *increased* civic engagement and green behavior.

## Method

### Sampling and data collection

To set the study's target sample size, we conducted a power analysis for a multiple linear regression analysis (G-Power 3.1.9.2 software)^46^. This indicated a sample size of 500 people is needed to achieve 0.80 power to detect a medium effect size (0.15).

To recruit respondents, we used Amazon's CloudResearch web survey software platform. CloudResearch maintains a nationally representative web panel of pre-recruited participants known as "Prime Panels.” To ensure enough highly engaged persons were recruited to sustain the analysis, we included the following yes or no screening question: “Have you spent five hours or more during the prior month working paid or unpaid on environmental protection issues or in the field of environmental conservation?" The first 250 respondents within each category of responses (i.e., yes and no) were invited to complete the survey to ensure a final sample size of 500. CloudResearch also used quotas to ensure that the respondents broadly matched census templates to be representative of the American population. Panel users were at least 18 years of age and based in the United States.

Respondents filled out a Qualtrics survey measuring their self-reported formative experiences, civic engagement, and green behavior as well as select demographic characteristics described below. Participants filled out the survey in one sitting. It took an average of ten minutes to complete the survey. To control for bots, we introduced a reCAPTCHA service from Google. Data cleaning and analysis occurred in Stata IC 14.2.

All methods were carried out in accordance with relevant guidelines, and informed consent was obtained from all subjects. This research was approved by the Institutional Review Board at the University of Colorado Boulder under protocol no. 19-0813.

### Measures

Table 1 provides descriptive statistics and Cronbach’s alpha scores for study variables. Table S2 provides information about each variable and its source.

**Table 1:** Description of key study variables and descriptive statistics.

Variable—Alpha included (if scale)	Description	Analytical sample (n = 449)		Population (census)	
		Mean	SD (range)	Mean	SD (range)
Age (years)	Mean age in years of survey respondent	41	15.89 (18–95)	38.4	(< 5 to 85 +)
Sex	% of respondents of each sex	50% female 50% male		51% female 49% male	
Education	% of respondents with highest degree of education	41% = HS or less 21% = Some College 27% = College Grad 11% = Professional		35% = HS or less 22% = Some College 25% = College Grad of Higher	
Political affiliation	% of respondents per political affiliation	47% democrat 36% republican 18% independent		^^^^50% democrat 42% republican 8% independent	
Gross household income	% of respondents per income bracket	13% = up to $19,999 13% = $20,000—$29,999 16% = $30,000—$39,999 12% = $40,000—$49,999 19% = $50,000—$74,999 27% = $75,000 or more		9% = up to $19,999 7% = $20,000–$29,999 8% = $30,000–$39,999 7% = $40,000–$49,999 17% = $50,000–$74,999 14% = $75,000 or more	
Children (Y/N)	% of respondents with children	63% = no children 37% = 1 child or more		^^^59% = no children 41% = 1 child or more	
Vote in last election (Y/N)	% of respondents voting status for the last election	71% = yes 29% = no		^#^46.6% = no 53.4% = yes	
Climate change belief	% of respondents’ belief in climate change [0] = Non-Believers/[1] = Believers	79% = belief 21% = belief		69% = belief ^^^^^31% = nonbelief	
Experiences in nature as a child (Alpha = 0.72)	Experiences in nature	3.75	0.890 (1–5)		
Travel Experiences as a child*Index measure no alpha calculated	Travel experiences	1.31	0.718 (0–2)		
Trauma as a Child* Index measure no alpha calculated	Trauma as a child	2.14	1.679 (0–8)	.	
Green behavior (Alpha = 0.90)	Green behavior measure	1.28	0.565 (0–2.875)		
Civic engagement (h)	Estimated number of hours a month spent on engagement work related to environmental protection or conservation, includes work done for volunteer or pay	29	45.88 (1–400)		

*Mean is reported unless another statistic is indicated in the description.

^^^Statista.com.

^#^2018 Voter Turnout: U.S Census Bureau: Current Population Survey Voting and Registration Supplements: 2014–2018.

^^^^Based on Pew Research Estimates of Registered Voters % that lean Democrat and Republican Per Estimated 2017 Voter Turnout Estimates: Report: Trends in Party Affiliation Among Demographic Groups.

^^^^^Based on an NBC National Poll Climate Change in the American Mind conducted in March 2018.

^1^Census Data was obtained from the 2018 American Community Survey Estimates Tables.

^2^Descriptives include outliers.

#### Environmental engagement

*Environmental engagement* comprised two measures: *civic engagement* and *green behavior*. Civic engagement was measured with the question: About how many hours a month do you spend engaged in work that is related to environmental protection or conservation? This may include work that is done as a volunteer or for pay. To generate an equal distant ordinal variable, we converted the continuous variable into quintiles. To measure green behavior, we used the 24-item green behavior scale used by Whitmarsh and O’Neill^47^ which ranged from 0 = never adopted to 3 = Always performed or very recently performed an action (depending on the question, see Table S2).

#### Formative experiences

We asked participants to answer questions about experiences in nature (4 items, 1 = strongly disagree to 5 = strongly agree), travel experiences (2 items, 0 = no and 1 = yes), trauma as a child (8 items, 0 = no and 1 = yes). Please note for the trauma index we include a poverty item based on the logic employed by scholars who categorize neglect or the “absence of provision of basic needs such as food or a safe home environment: as existing under the broad category of childhood and adolescent trauma”^43,48^. Moreover, scholars have shown how growing up poor can be traumatic for some and may be associated with social rejection^49^. For experiences in nature, the scale was averaged to create a composite score. The items for travel experiences (out of 2) and trauma as a child (out of 8) were summed to create a single score for travel experiences and trauma as a child representing the total number of experiences the respondent had as a child.

#### Demographics

We asked respondents several demographic questions about age, sex, education, political affiliation, and gross household income. We also asked if they voted in the last election and if they have children. To collect political affiliation data, we used an approach used by Eby and colleagues^50^ in which the authors use the following codes: Democrat (1), Independent but lean Democrat (2), Republican (3), Independent but Lean Republican (4), Independent no leaning (5), no affiliation. Those who selected options 1, and 2, were classified as Democrats and those who selected options 3 and 4 were classified as Republicans; others were classified as either Independent or missing data.

### Analytical sample

502 participants completed the survey. Two cases where there were obvious data entry errors were excluded. Specifically, one person was removed because they reported their age as four years old. Another person was removed because they reported having engaged in environmental protection work for more than 700 h in a month, which exceeded the number of hours in an average month. Fifty-one cases were also removed because they were missing data on one of the key variables or covariates as described in Table 1. The final analytical sample was 449. Table 1 summarizes the complete case or analytical sample.

## Results

Respondents were, on average, moderately engaged. The mean green behavior score was 1.28 (SD 0.57), suggesting they performed the selected actions a little more than occasionally (for routine behaviors) or 5 + years ago (for infrequent behaviors). Civic behavior scores indicated that the average respondent spent 29 h a month on environmental work (SD 45.88). Although this data point suggests a very high level of engagement, the distribution was skewed by a small proportion of respondents, approximately 11% that were highly engaged; possibly reflecting a subset of full-time environmental professionals. Approximately 51% of the sample reported less than 1 h of environmental engagement per month.

With respect to the SLE variables, the average respondent scored a 3.75 (SD 0.89) on the experiences in nature measure (out of 5) and a 1.31 (SD 0.72) on travel experiences (out of 2). On average, participants reported 2.14 traumatic experiences in childhood (SD 1.68). The most common traumatic experiences reported were the loss of a parent, sibling, or partner, personal health issues, sexual harassment, sexual assault, and bullying encounters as a child or as an adult.

### Civic engagement

Table 2 summarizes two ordered logistic regression models predicting civic engagement as a function of demographic and formative experience. We use an ordinal model due to the highly skewed distribution of the outcome variable. Model 1 is the baseline model with only demographic covariates, which explained approximately 4.4% of the variance in civic engagement. In Model 2 we added in the formative predictors to the existing set of covariates. Model 2 yielded a slightly higher pseudo r-squared (5.7%) than Model 1. Model 2 results show a positive relationship between childhood trauma and civic engagement. A one unit increase in trauma was associated with a 22% higher odds of scoring in a higher quintile of civic engagement (OR 1.22, p < 0.01). Experiences in nature and travel experience as a child were not significant correlates of civic engagement.

**Table 2:** Ordered logistic regression predicting civic engagement.

	Model 1		Model 2	
	OR	SE	OR	SE
**Fixed effects**				
Cut one intercept of ordinal measure—little engagement	0.714	0.448	1.620	0.621
Cut two intercept of ordinal measure—modest engagement	1.255	0.451	2.178	0.625
Cut three intercept of ordinal measure—very engaged	1.971	0.457	2.911	0.633
Cut four intercept of ordinal measure—extremely engaged	2.916	0.470	3.874	0.645
**Demographic**				
Age *reference category* = *Gen Z* (18–22)				
Millennials (23–38)	0.775	0.300	0.765	0.298
Generation X (39–54)	0.737	0.294	0.770	0.308
Baby Boomers/Silent Generation (55 +)	0.389*	0.168	0.439	0.191
Sex *reference category* = *female*				
Male	1.108	0.208	1.011	0.194
Education *reference category* = *high school graduate or less*				
Some college	0.824	0.212	0.771	0.201
College grad	2.006**	0.498	1.964**	0.495
Professional or doctoral degree (JD, MD, Ph.D.)	1.257	0.416	1.275	0.427
Political affiliation *reference category* = *democrat*				
Republican	0.747	0.154	0.767	0.160
Independent	0.893	0.254	0.876	0.252
Gross household income *reference category* = ≤ *$19,999*				
$20,000–$29,999	1.939	0.749	1.915	0.749
$30,000–$39,999	1.555	0.593	1.563	0.600
$40,000–$49,999	2.563*	1.021	2.391*	0.963
$50,000–$74,999	2.276*	0.832	2.257*	0.839
$75,000 or more	1.744	0.641	1.763	0.129
Vote in last election *yes/no*	1.550	0.379	1.583	0.391
Children *yes/no*	1.171	0.117	1.154	0.117
**Formative experiences**				
Experiences in nature as a child			1.120	0.132
Travel experiences child			1.059	0.154
Trauma as a child			1.222***	0.069
**Model fit**				
Pseudo R^2^	0.044		0.057	
BIC	1302.072		1304.016	
N	449		449	

Unstandardized coefficients reported *p < 0.05, **p < 0.01, ***p < 0.001.

*OR* odds ratio, *SE* standard error.

### Green behavior

Table 3 summarizes a series of multiple linear regression models using the same set of variables to predict green behavior as in Table 2. Model 1 is again the baseline model with only demographic covariates and in Model 2 we added formative experiences. The covariates in Model 1 explained 13 percent of the variance in green behavior. Including the three SLE variables in Model 2 resulted in much improved explanatory power (*R*^2^ = 29%). All formative experiences were positively and significantly associated with green behavior, with effect sizes within the small to moderate range. Among these, childhood trauma was the strongest predictor (β = 0.24, p < 0.001). The second-largest effect was experiences in nature as a child (β = 0.21, p < 0.001), followed by travel experiences (β = 0.16, p < 0.001).

**Table 3:** Regression predicting green behavior.

	Model 1		Model 2	
	β	SE	β	SE
**Fixed effects**				
Green behavior intercept	1.057***	0.119	0.198	0.152
**Demographic**				
Age *reference category* = *Gen Z* (18–22)				
Millennials (23–38)	− 0.123	0.108	− 0.080	0.098
Generation X (39–54)	− 0.138	0.111	− 0.084	0.101
Baby boomers/silent generation (55 +)	− 0.102	0.118	0.015	0.109
Sex *reference category* = *female*				
Male	0.009	0.053	− 0.015	0.048
Education *reference category* = *high school graduate or less*				
Some college	0.067	0.071	0.026	0.064
College grad	0.135*	0.071	0.096	0.065
Professional or doctoral degree (JD, MD, Ph.D.)	0.074	0.094	0.058	0.084
Political affiliation *reference category* = *democrat*				
Republican	− 0.125*	0.059	− 0.100*	0.053
Independent	− 0.039	0.077	− 0.019	0.070
Gross household income *Reference category* = ≤*$19,999*				
$20,000–$29,999	0.051	0.101	0.028	0.092
$30,000–$39,999	0.114	0.098	0.123*	0.089
$40,000–$49,999	0.099	0.106	0.058	0.097
$50,000–$74,999	0.107	0.096	0.090	0.088
$75,000 or more	0.232	0.097	0.199**	0.088
Vote in last election *yes/no*	0.166**	0.067	0.174***	0.061
Children *yes/no*	0.070	0.029	0.052	0.026
**Formative experiences**				
Experiences in nature as a child			0.211***	0.029
Travel experiences child			0.162***	0.036
Trauma as a child			0.235***	0.014
**Model fit**				
R^2^	0.127		0.288	
BIC	804.084		731.057	
N	449		449	

*β* standardized beta coefficients, *SE* standard error.

*p < 0.05, **p < 0.01, ***p < 0.001.

## Discussion

We looked at how formative experiences correlate with engagement among a sample of American adults. We put forth two research questions: (1) Are SLE's (e.g., experiences in nature, travel experiences, and childhood trauma) a correlate of civic engagement measured in hours per month for a sample of American adults, and (2) Are SLE's a correlate of green behavior for this sample? Drawing on the SLE framework, we hypothesized that more formative experiences would correlate with increased civic engagement and green behavior. We find that childhood trauma was a significant positive correlate of civic engagement, and all three formative experiences were significant positive correlates of green behavior. Consequently, we find evidence in support of both hypotheses.

### Experiences in nature

We find that experiences in nature was associated with green behavior. This finding aligns with earlier literature^2,51^ and supplies quantitative evidence for why experiences in nature is consistently ranked as the most prominent of the formative experiences within the SLE literature^3,5,18,52^. This finding also aligns with earlier literature which shows how exposure to nature is positively associated with greater engagement^53^. As people continue to grapple with climate change and other environmental protection issues, the importance of experiences in nature remains salient. What our finding adds to the literature is that childhood experiences in nature may be more strongly associated with green behavior as compared to civic engagement.

### Travel experiences

Travel experiences was a significant positive predictor of green behavior but not of civic engagement. Previous studies show that travel is a potentially transformative endeavor as it facilitates learning about new things^27^. It can also make people feel more attuned to problems that transcend borders, such as climate change^28,54^. What our finding adds to the literature is that travel may be more strongly associated with green behavior as compared to civic engagement.

### Trauma as a child

We find that trauma as a child was positive and significant for both civic engagement and green behavior. Trauma as a child also had the largest effect size in comparison to the other formative experiences for green behavior. Prior evidence suggests that traumatic events experienced earlier in life like the loss of a family member or experiences of physical and sexual violence are positively associated with empathy^34,37–39^. We also know there is a positive association between empathy and green behavior^40,41^.

Empathy, in the broadest sense, involves taking the perspective of another. It has been described at times as an emotion and at other times as a process^55,56^ and can also be understood as a “phenomenological experience” that is grounded on the similarity of experiences^56^. We also know that humans can feel empathy towards the environment^40,41^. It may be that people who have experienced trauma may feel empathy towards the environment because they perceive it as vulnerable and therefore in need of protection. Others may relate to the experience of harm or injury observed in environmentally degraded spaces.

These data suggest that childhood trauma may influence environmental decisions in later life. Similar evidence from political science shows that childhood trauma correlates with political ideology in later life, specifically that it moderates the relationship between the personality trait of openness and political ideology^57^.

In sum, our findings show that childhood trauma predicts both civic engagement and green behavior, while childhood experiences in nature and childhood travel experiences predict green behavior but not civic engagement. Prior research may explain this pattern. Stern and colleagues distinguish between two spheres of environmental engagement: public and private^17^. Private sphere behavior includes personal or household behaviors, for example, the decision to recycle or take shorter showers. In comparison, public sphere behaviors involve environmental citizenship behaviors; for example, writing to an elected official or donating to an environmental organization. In this study, we used two different outcome measures to capture both private (green behavior) and public sphere behavior (civic engagement). Our findings show that childhood trauma is associated with both public and private sphere behaviors. A possible explanation for this finding is that childhood trauma is so encompassing and psychologically scarring that it affects public and private sphere behaviors. In comparison, the other formative experiences may not have the same resonance and significance to a person as experiencing childhood trauma does. Therefore, childhood experiences in nature and travel may be associated with private sphere behaviors but not rise to the level of public sphere behavior.

Some individuals have gone on to launch initiatives or found organizations after they experienced trauma. For instance, Mothers Against Drunk Driving (MADD) was founded after a mother lost her child to drunk driving in 1979^58^. Moreover, researchers have shown that experiencing a traumatic event is linked to volunteer work with a larger number of organizations^33^ partly because the act could be a coping mechanism for the trauma^32,33^. A more nuanced understanding of childhood trauma is needed, especially its relationship with pro-social outcomes like empathy, altruism, compassion, and perspective-taking and how these factors in turn motivate a desire to engage in environmental issues or pursue environmental vocations. In addition, more research is needed on how formative experiences influence public and private behaviors, as different formative experiences appear to be associated with each sphere.

## Limitations

This research is not without limitations. First, we used self-reported data, which is known to have reliability and validity issues. For instance, survey participants may have reported socially desirable answers as opposed to fully factual responses. Future iterations of this research should consider alternative measurement strategies. This might include using an observational green behavior measure rather than a self-reported one. Second, this was a cross-sectional study. It offers some indication of how the relationship between SLE’s, civic engagement, and green behavior operate. However, these data do not permit causal inference.

Despite these limitations, we add to the SLE literature because we look beyond environmental adverse experiences like experiencing floods and hurricanes^2,30^ to personal trauma experienced as a child and how this personal trauma positively correlates with environmental engagement. In so doing, we add to the body of work about precursors to engagement.

## Study implications and future directions

In the face of pressing environmental issues, engagement remains low in the United States^1^. What drives engagement thus is an important issue to investigate. Our research advances the study of environmental engagement in several ways. We find evidence that certain formative experiences during childhood inspire environmental engagement later in life. Like others, we see that time spent in nature correlates with private-sphere pro-environmental behaviors in adulthood. This adds additional evidence for the conclusion that nature experiences support emotional connections with or curiosity about the natural environment that may shape later behavior. These findings reinforce the importance of creating opportunities for children to learn and play in natural spaces^59^. An example of such an initiative is *The Get to Know Program (Program)* which began in 2000. The Program’s main aim was to engage elementary and high school youth in nature-based activities. Youth in the program observed wildlife, hiked, took part in a Natural Treasure Adventure, and made art and other mixed media that reflected their time in nature^59^. Furthermore, the finding that childhood trauma has an even stronger relationship with environmental engagement is more novel and somewhat surprising. Given the large literature suggesting that childhood trauma sometimes undermines pro-social behavior towards other humans, these data indicate the need for further research. This finding may reveal something about when or why people empathize with the environment as well as the significance of empathy for private and public-sphere pro-environmental action. We did not measure empathy or other forms of emotional engagement in this study, but future research should consider these variables in relation to early life experiences.

Our findings also corroborate other research demonstrating that trauma can be associated with pro-social outcomes^32,33,36^. Future studies should consider whether the target of pro-social behavior (e.g., other humans vs. non-human nature) moderates the effects of trauma on behavior. Finally, our results also suggest that many American adults who engage environmentally have overcome adversity. Scholars have documented variables that can support someone after they have experienced trauma, such as social support^33,43^. Our findings indicate a possible need for more trauma-informed resources and social support programs for those who engage.

## Conclusion

We examine how formative experiences correlate with the civic engagement and green behavior patterns of a nationally representative panel of American adults. We find that among the formative experiences used to predict civic engagement and green behavior, it was trauma as a child that was most important, which had a small to moderate positive association with environmental engagement. Our key finding: trauma as a child is a positive predictor of civic engagement and green behavior. Childhood experiences in nature had the second-largest effect size in comparison to the other formative experiences for green behavior. The finding that experiences in nature positively predicts green behavior corroborates much of the existing literature using data from a representative sample in the U.S. On the other hand, the finding that childhood trauma also predicts engagement is more novel and points to the possible role of empathy as a precursor to environmental engagement.

Scholars continue to call for more research on environmental action and impact with an eye toward broadscale policy change^60^. Effective environmental action can only be understood once we study the drivers behind what motivates the American public to protect the environment. Consequently, we have attempted to gain insight into the perspective of the American adult public. This information will help us get to know better the view of the American adult public regarding three significant life experiences, which is a needed first step in understanding the audiences that will be critical to environmental protection.

## Supplementary Information

Supplementary Tables.

## Data Availability

The datasets generated during the current study are not publicly available but are available from the corresponding author upon reasonable request.
