# Author Correction: Childhood trauma and other formative life experiences predict environmental engagement

**DOI:** 10.1038/s41598-023-27834-7

**Published:** 2023-01-16

**Authors:** Urooj S. Raja, Amanda R. Carrico

**Affiliations:** 1 School of Communication, Loyola University Chicago, Chicago, IL USA; 2 Department of Environmental Studies, The University of Colorado Boulder, Boulder, CO USA

Correction to: *Scientific Reports* https://doi.org/10.1038/s41598-022-24517-7, published online 01 December 2022

The Funding section in the original version of this Article was incomplete.

“This work was partly supported by a research grant from the Department of Environmental Studies at the University of Colorado at Boulder to offset the cost of data collection. Publication of this article was funded by the University of Colorado Boulder Libraries Open Access Fund.”

now reads:

“This material is partially based upon work supported by the National Science Foundation Graduate Research Fellowship Program under Grant No. (NSF grant number DGE 1650115). Any opinions, findings, and conclusions, or recommendations expressed in this material are those of the author(s) and do not necessarily reflect the views of the National Science Foundation. We would also like to thank the Center for Research Data and Digital Scholarship Center (CRDDS) at the University of Colorado Boulder and Nickoal Eichmann-Kalwara for fellowship support, the Graduate School of Arts and Science at the University of Colorado, the Center to Advance Research and Training in the Social Sciences, and the Department of Environmental Studies for grants at the University of Colorado to offset the cost of data collection. Publication of this article was funded by the University of Colorado Boulder Libraries Open Access Fund.”

The original Article has been corrected.

