# Supplementary material for: Childhood trauma and other formative life experiences predict environmental engagement
Source: Sci Rep. 2022 Dec 1;12:20756. doi: 10.1038/s41598-022-24517-7 (PMC9715940; doi:10.1038/s41598-022-24517-7)
Supplement: Supplementary file 1 — Supplementary Tables. [file 41598_2022_24517_MOESM1_ESM.docx]

APPENDIX (Supplementary Materials)

**Table S1 Correlations of Key Variables in Models**

|  |  | 1 | 2 | 3 | 4 | 5 | 6 | 7 | 8 | 9 | 10 |
| --- | --- | --- | --- | --- | --- | --- | --- | --- | --- | --- | --- |
| **Variable Type** |  |  |  |  |  |  |  |  |  |  |  |
| *Demographic* | 1.Age | -- |  |  |  |  |  |  |  |  |  |
|  | 2.Sex | 0.027 | -- |  |  |  |  |  |  |  |  |
|  | 3.Education | 0.063 | 0.051 | -- |  |  |  |  |  |  |  |
|  | 4.Political Affiliation | 0.012 | -0.015 | -0.158 | -- |  |  |  |  |  |  |
|  | 5.Gross Household Income | -0.069 | 0.079 | 0.430 | -0.126 | -- |  |  |  |  |  |
|  | 6. Vote | 0.302 | 0.045 | 0.205 | -0.248 | 0.192 | -- |  |  |  |  |
|  | 7. Children (Y/N) | -0.182 | 0.044 | 0.048 | -0.044 | 0.194 | 0.068 | -- |  |  |  |
| *Formative Experiences* | 8. Experiences in Nature as a Child | -0.129 | -0.050 | 0.120 | -0.083 | 0.133 | -0.009 | 0.143 | -- |  |  |
|  | 9. Travel Experiences as a Child | -0.136 | 0.043 | 0.189 | -0.097 | 0.218 | -0.002 | 0.047 | 0.354 | -- |  |
|  | 10. Trauma as a Child | -0.170 | 0.104 | -0.072 | 0.025 | -0.060 | -0.085 | 0.038 | 0.129 | 0.183 | -- |
|  |  | **Numbers following the terms in the column correspond to the numbers in the rows* | | | | | | | | | |

**Table S2 Items Used in Survey and Source Adapted from**

|  | | |  |  |  |
| --- | --- | --- | --- | --- | --- |
| **Construct** | | | **Question** | **Item Options** | **Adapted From/Source** |
| **Formative Experiences** | | |  |  |  |
| Experiences in Nature Child | | [1] I spent a lot of time in nature during my childhood (age 5 to 12 years). [2] During my childhood (age 5 to 12 years), I spent time in nature accompanied by my friends/youth groups. [3] During my childhood (age 5 to 12 years), I spent time in nature accompanied by my teachers.  [4] During my childhood (age 5 to 12 years), I spent time in nature accompanied by my family. | | (1=strongly disagree - 5= strongly agree) | Kals, Schumacher, & Montada, 1999 |
| Travel Experiences Child | The next set of questions will ask you about your travel experiences as a child (between the ages of 5 and 12) and as an adult. Please answer either yes or no.  [1] I had a memorable travel experience(s) as a child. [2] I traveled for educational purposes as a child (for example, study abroad programs, school-related events, training programs, and or instructional or research field trips? | | | (0=No 1= Yes) | Kim 2018; Kals, Schumacher, & Montada, 1999 |
| Trauma as a Child | Have you experienced any of the following, as a child (between the ages of 5 and 12): -  [1] Natural disaster (for example flood, hurricane, tornado, earthquake) [2] Transportation accident (for example car accident, boat accident, train wreck, plane crash) [3] Exposure to toxic substance (for example dangerous chemicals, radiation) [4] Physical or sexual assault (for example being attacked, hit, slapped, kicked, beaten up) [5] Combat or exposure to a warzone (in the military or as a civilian) [6] Life-threatening illness or injury [7] Were you bullied (for example called mean names AND/OR made fun of or teased in a hurtful way?) [8] As a child, did your family receive assistance from an income based-assistance program (example-Aid to Families with Dependent Children, Food Stamps, Supplemental Nutrition Assistance Program, or Medicaid). | | | (1=yes; 0= no, index) | Weathers, Blake, Kaloupek, Marx, & Keane, 2013 [Items 1-6] Breivik & Olweus, 2015 [Item 7] Eckenrode, Laird, & Doris, 1993 [Item 8] DSM |
| Belief in Climate Change | Thinking about the causes of climate change, which, if any, of the following best describes your opinion? | | | [**0**] =  [] Climate change is entirely caused by natural processes. [] Climate change is mainly caused by natural processes. [] I think there is no such thing as climate change.  **[1**] =  [] Climate change is partly caused by natural processes and partly caused by human activity. [] Climate change is mainly caused by human activity. [] Climate change is entirely caused by human activity. | Spence et al. 2012 |
| Green Behavior | Please indicate the last time you took this action (if at all) –  [1] Installed insulation products in your home" [2] Bought or built an energy-efficient home" [3] Installed a more efficient heating system" [4] Installed a renewable energy system (e.g., solar panels, wind turbine) in your home" [5] Purchased renewable energy offsets or credits as part of your energy service" [6] Bought a low-emission vehicle (e.g., hybrid, electric, biofuel, less than 1.4 L engine)" [7] Bought a product to save water (e.g., low-flush toilet, low flow showerhead ) [8] Turn off lights you’re not using [9] Drive economically (e.g., braking or accelerating gently) [10] Walk, cycle or take public transport for short trips (i.e., trips of less than 3 miles) [11] Use an alternative to traveling (e.g., shopping online) [12] Share a car ride with someone else (carpool) [13] Cut down on the amount you fly [14] Buy environmentally friendly products [15] Eat food which is organic, locally grown or in season [16] Avoid eating meat [17] Buy products with less packaging [18] Recycle [19] Reuse or repair items instead of throwing them away [20] Compost your kitchen waste [21] Save water by taking shorter showers [22] Turn off the faucet while you brush your teeth [23] Write to your political representative about an environmental issue [24] Take part in a protest about an environmental issue | | | Items 1-7 (never (0), 5 or more years ago (1), 1–3 years ago (2), in the last year (3)   Items 8-24( never (0), occasionally (1), often (2), always (3).) | Whitmarsh and O’Neill, 2010 |
|  |  | |  |  |  |
